# Identification of Speech Characteristics to Distinguish Human Personality of Introversive and Extroversive Male Groups

**DOI:** 10.3390/ijerph17062125

**Published:** 2020-03-23

**Authors:** Jangwoon Park, Sinae Lee, Kimberly Brotherton, Dugan Um, Jaehyun Park

**Affiliations:** 1 Department of Engineering, Texas A&M University-Corpus Christi, Corpus Christi, TX 78412, USA; Jangwoon.Park@tamucc.edu (J.P.); kimberly.brotherton@yahoo.com (K.B.); Dugan.Um@tamucc.edu (D.U.); 2 Department of English, Texas A&M University-Corpus Christi, Corpus Christi, TX 78412, USA; Sinae.Lee@tamucc.edu; 3 Department of Industrial & Management Engineering, Incheon National University, Incheon 22012, Korea

**Keywords:** personality, speech, reaction time, sound pressure, pitch, linguistics, human-robot interaction, user experience, usability

## Abstract

According to the similarity-attraction theory, humans respond more positively to people who are similar in personality. This observation also holds true between humans and robots, as shown by recent studies that examined human-robot interactions. Thus, it would be conducive for robots to be able to capture the user personality and adjust the interactional patterns accordingly. The present study is intended to identify significant speech characteristics such as sound and lexical features between the two different personality groups (introverts vs. extroverts), so that a robot can distinguish a user’s personality by observing specific speech characteristics. Twenty-four male participants took the Myers-Briggs Type Indicator (MBTI) test for personality screening. The speech data of those participants (identified as 12 introvertive males and 12 extroversive males through the MBTI test) were recorded while they were verbally responding to the eight Walk-in-the-Wood questions. After that, speech, sound, and lexical features were extracted. Averaged reaction time (1.200 s for introversive and 0.762 s for extroversive; *p* = 0.01) and total reaction time (9.39 s for introversive and 6.10 s for extroversive; *p* = 0.008) showed significant differences between the two groups. However, averaged pitch frequency, sound power, and lexical features did not show significant differences between the two groups. A binary logistic regression developed to classify two different personalities showed 70.8% of classification accuracy. Significant speech features between introversive and extroversive individuals have been identified, and a personality classification model has been developed. The identified features would be applicable for designing or programming a social robot to promote human-robot interaction by matching the robot’s behaviors toward a user’s personality estimated.

## 1. Introduction

The digital footprints people leave on online sites can be vigorously used to customize advertisement for each individual while they are online. A recent study by Dr. Sandra Matz at Columbia Business School demonstrated that extroverted individual preferred simple images and images that featured people, while more open-minded individuals favored pictures with no people and with cool colors like blue and black [1]. Therefore, capturing a customer’s personality and emotions will eventually shape the advertisement industry to a level unprecedented so far. In addition, the more the users use the robot or AI devices, the better the chance of success of the advertisement. Therefore, the question as to how to increase the usability of such devices is another important domain question in Human-Robot Interaction (HRI).

Humans engage daily in many verbal interactions, ranging from small talk to purposeful conversations. The topic, length, sound pressure, and pitch of these conversations varies, as with the speaking and conversational style of individuals who participate in the interaction. Throughout an interaction, humans can infer multiple layers of information being conveyed, including the meaning beyond the literal level, or the conversational implicature [2]. Additionally, humans carry out everyday conversations with minimal effort, exhibiting subconscious knowledge in complex turn-taking system [3]. Humans can also easily identify the tone of voice, which is then used as one of the cues to infer the emotional state and personality traits.

What facilitates the humans’ interactions with each other is the additional ability to modify our behavior according to the context of speech as well as the interlocutors, along with inferring various information conveyed during the conversation. The modification of speech and language use on an individual level, also known as stylistic variation, has been well discussed in the field of linguistics. For example, Labov (1972) [4] observed that people adjust their pronunciations depending on how formal speaking situations are. Bell (1984) [5] proposed the effect of the audience after demonstrating how people adjust their speech depending on who they are talking to. Bell’s (1984) proposal is connected to the Communication Accommodation Theory [6], the framework of which seeks to explain communicative variation found within an individual interaction in various social functions. The recent development in linguistics also points to the type of intra-speaker variation (also known as Speaker Design) that reflects speaker agency in adopting different styles of speech in order to establish a particular stance or a persona [7,8,9].

The assumption here is that certain styles of speech found among those who speak the same language are socially recognized when adopted. In other words, there are associations between a group of people and their way of speaking. When humans interact with each other, these associations can be utilized in order to infer information about the person. While humans can infer this information including personality traits, this is yet to be the case for machines [10]; accurate and reliable identification of personality traits remains challenging for machines.

According to the similarity-attraction theory, humans respond more positively to people who have a similar personality [11]. Similar relational interaction has also proved to be true between humans and robots, especially for socially assistive robots (SAR) [12]. SARs are currently being developed to work as caregivers alongside doctors, nurses, and physical therapists [13]. For example, SARs aid emotional and mental therapy for children with autism [14,15], as well as providing companions in nursing homes [15,16]. Because the personal characteristics of patients are varied, a SAR needs to be versatile in employing different interactional strategies so that it can effectively assist differing patients with their rehabilitation efforts. To do that, a SAR needs to identify a patient’s personality during patient-robot interaction and modify its aid strategy per the patient personality.

Many studies have identified positive effects of matching the robot-human personality on therapeutic, entertaining, and driving tasks. For example, Andrist et al. (2015) observed that the amount of time spent during a therapeutic task significantly increased upon matching the SAR’s gaze behavior to the user’s personality. Additionally, users interacting with a gaze-matching SAR completed their tasks more frequently than those interacting with a non-gaze-matching SAR. Similarly, Goetz et al. (2003) [17] found that a robot’s demeanor (friendly vs. serious) in an entertaining task significantly influences users’ perceptions of a robot (e.g., friendly, playful, and witty) and their willingness to comply with the robot’s instruction. Nass et al. (2005) [18] showed that pairing the voice of the car (e.g., energetic voice) with the driver’s emotion (e.g., happy) had a positive effect on the perceived attention to the road.

There are several studies that identified linguistic features that may be associated with aspects of human personality. For example, Jurafsky et al. (2009) [19] note that certain linguistic and interactional features—namely, laughter, speech rate, and pitch range—can be used in successfully detecting particular elements of interactional style, such as friendliness or flirtatiousness. Ranganath et al. (2009) [20], who conducted a separate study on the same dataset used in Jurafsky et al. (2009), additionally report that their detection system using various prosodic, interactional, and lexical features can identify speakers’ flirtatious intent with 71.5% accuracy, even outperforming the human perception.

Prosodic and acoustic features are shown to be particularly useful in detecting personality traits, as both Mohammadi and Vinciarelli (2012) [21] and Polzehl et al. (2010) [22] show that pitch range, speech rate, intensity, loudness, formants, or spectrals can predict the elements of the Big Five. This does not necessarily mean, however, that other aspects of language, such as lexical choices or the amount of words used, are less indicative. For instance, Beukeboom et al. (2013) [23] found that the level of language abstraction differed between extraverted Dutch speakers and introverted Dutch speakers, in which extraverts exhibit verbal styles that include more markers of abstraction. In examining the text messages among extroverts and introverts, Gill and Oberlander (2002) [24] found that extroverts produce more words in text messages than introverts.

A number of previous studies, including some mentioned above, observed the likely association between extroverts and verbal fluency [25]. For example, many studies documented the tendency to speak more among extroverts [26,27,28,29]. Similarly, the tendency to pause less and shorter during speech among extroverts has been observed [30,31], though this particular tendency may not hold true cross-culturally [32,33]. The current study attempts to continue this line of inquiry by exploring various characteristics of speech in relation to the speaker extroversion/introversion.

However, many speech data in the previous studies were collected in the naturalistic conversations (spontaneous speech) without restricting the conversation topics, which might increase the uncertainty of personality effects on the speech data. Depending on the conversational topics, people often speak in different speed, employing different acoustic and lexical features [34]. To explain the effects of personality on speech characteristics, the conversation topics need to be controlled. The current study addressed this by controlling the conversation topics, mainly by using the same questions in the same scenario so that the nuisance effects of a conversation topic on the data were eliminated. Such a controlled experimental design protocol would yield an identification of the pure effects of personality on speech, sound, and lexical characteristics.

## 2. Methods

### 2.1. Participants

Twenty-four university male students in their 20 s who are native speakers of American English participated in this study. To recruit similar numbers of introversive and extroversive male participants, a pre-screening process was completed in which we checked a potential participant’s Myers-Briggs Type Indicator (MBTI) personality via an online tool (16 Personalities, NERIS Analytics Limited, United Kingdom). Along with the Big Five test, the MBTI is one of the most popular tools used in studying personality [35]. The test is taken in a questionnaire format, and identifier letters are assigned to the 16 personality types, where eight types belong to introverts, and the other eight types belong to extroverts. For example, if a participant was classified as “INFJ” out of the 16 personality types (the first letter “I” indicates introversion, the second letter “N” indicates intuition, the third letter “F” indicates feeling, and the last letter “J” indicates judging), then he/she was classified as an introversive person; if a participant was classified as ENFJ (the first letter “E” indicates extraversion), then the person was classified as an extroversive person. In this study, we recruited 12 introversive and 12 extroversive males based on their MBTI personality types. Most of the participants were university students in their 20 s. Table 1 shows the MBTI classes of the 24 participants.

### 2.2. Apparatus

To obtain participants’ speech, the Walk-in-the-Woods questionnaire is prepared. Their responses were recorded with a high definition audio recorder. The Walk-in-the-Woods relational psychology test was performed by a moderator ensuring the same test conditions for each participant: types of questions, interview duration, and the pace of conversation, etc. The reasons why we chose the Walk-in-the-Woods questions are because (a) it is efficient for this study due to its brevity, and (b) the answers participants are asked to provide are short, but cognitively demanding, since the questions are designed to reflect the participant’s emotional, psychological, and cognitive process. This test consists of eight questions (see the list below). These questions indicate relevance to values that the data pool subjects deem important in their personal lives [36]. The eight questions are the following:

(1) Picture yourself walking through a beautiful forest. The sun is out; there’s a perfect breeze. It’s just beautiful. Who are you walking with?
(2) As you continue in your walk through the forest, you come across an animal. What kind of animal is it?
(3) You come up to the animal. What does the animal do?
(4) You’re walking deeper into the woods, and you come to a clearing. There’s a house in the middle of the clearing. How big is it? Is it fenced in or no?
(5) You walk up to the door of the home, and it’s opened a bit. You enter and see a table. Describe what’s on the table.
(6) You finish looking around the house and leave out the back door. There’s a huge lawn, and in the center, there is a garden. In the garden, you find a cup. What is the cup made out of? What do you do with the cup?
(7) As you walk to the end of the garden, you find yourself at a body of water. What kind of body of water is it? A lake? River? Pond?
(8) You must cross this water to get home. How wet can you get?

### 2.3. Data Collection Procedures

The data was collected in a sound-controlled environment at a laboratory setup, at times that work best for each participant and a moderator. At the beginning of the experiment, the purpose was explained to the participants by a moderator. Their informed consent forms were obtained before the test. For each session, the moderator asked the eight questions from the aforementioned Walk-in-the-Woods questionnaire one by one. In order to reduce the effects of the moderator in the participants’ responses, the moderator was instructed to speak with a constant speed and loudness. The moderator was also instructed not to discuss topics other than asking the Walk-in-the-Woods questions. Each question item was followed by an answer, after which the moderator moved onto the next question item. Throughout the experiment, the moderator’s and participants’ voices were recorded. The sampling rate of the recorded audio was 48,000 data points per second. On average, the experiment took approximately five minutes to complete. This experimental procedure was approved by the Texas A&M University-Corpus Christi, Institutional Review Board (IRB, ID: 61-18).

### 2.4. Feature Extractions

The recordings were analyzed to quantitatively characterize the speech and sound features that might be relevant to human personality. The features extracted from the recordings include the following: (1) Averaged reaction time (ART) of all answers to the questions, (2) total reaction time (TRT) of all answers, (3) total speech time (TST) of all answers, (4) averaged pitch frequency (APF) of all answers, and (5) averaged sound power (ASP) of all answers. In this study, reaction time was defined as the silent period between the end of the moderator’s question and the beginning of the participant’s verbal response for each question. The beginning of the participant’s verbal response was defined as any sound response including a filler (e.g., um, well). Due to it being reported that the brain system of initiating a response would be different depending on the human personality [37], we presumed that the reaction time is related to the human personality.

**Figure 1:**
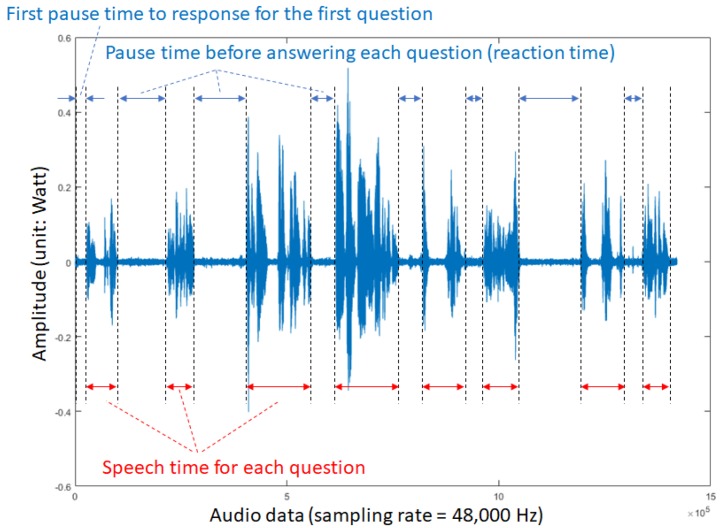
A plot of recorded audio of an introversive participant by using the matrix laboratory software (MATLAB).

The reaction times of the participants’ answers were taken from the recorded audio files. To analyze the reaction time of each participant, the moderator’s voice is removed from the recorded audio files. Then, each participant’s reaction time was measured with millisecond accuracy. Figure 1 illustrates a voice pattern of a participant with introversive nature. The horizontal axis indicates recorded data points with a sampling rate of 48,000 data per second, and the vertical axis indicates the amplitude of the recorded sound.

The audio data from each sample is recorded as stereo sound data. To calculate sound power, the audio data from the first channel is extracted. Next, the sound power is computed by Equation (1) for each answer. Then, the value of sound power for each answer is averaged to register an averaged sound power data for all participants. Another measurement utilized the speech analysis software Praat [37], which calculated the minimum pitch, maximum pitch, mean pitch, median, standard deviation, and audio duration for each audio file that contains only the participant’s voice (see the above figure).

(1) $$\mathrm{Averaged}\;\mathrm{sound}\;\mathrm{power}=\frac{\sum_{i=0}^{n}\sqrt{{\left(\mathrm{amplitude}\right)}_{i}{}^{2}}}{n}$$

In addition to these characteristics, responses to the questions were compiled to see the potential linguistic patterns for extroversion/introversion. The following was examined for this purpose: (1) total number of words (TNW) used in answering the eight questions, (2) total number of hedges (TNH) used in answering the eight questions (i.e., filler words), (3) total number of mitigators (TNM) used in answering the eight questions (i.e., words used to weaken the propositional force, such as “maybe”, “just”, or “I guess”), (4) total number of high-rising terminals (TNHRT) used in answering the eight questions (i.e., ending a declarative sentence with a rising intonation).

To conduct statistical testing on the extracted speech, sound, and lexical features, two-sample t-test method was employed to identify significant features with a significant level at 0.05. After the statistical testing for each speech, sound, and lexical feature, binary logistic regression models were developed to estimate two personality classes (introversive vs. extroversive) by incorporating the identified significant features.

## 3. Results

Table 2 shows descriptive statistics for the averaged reaction time, total reaction time, total speech time, averaged pitch frequency, and averaged sound power of the male participants for the introversive and extroversive groups. The mean ± standard deviation (SD), minimum, and maximum of the averaged reaction time are 1.2 ± 0.4 s, 0.6 s, and 2.2 s for the introversive group, and 0.8 ± 0.3 s, 0.4 s, and 1.3 s for the extraverted group. The mean difference of the averaged reaction time between the introversive and extroversive groups is 0.4 s and the statistical testing result (*p* = 0.010) indicates that the difference is significant between the two groups. Also, the mean ± SD, minimum, and maximum of the total reaction time are 9.4 ± 3.1 s, 4.5 s, and 15.6 s for the introversive group, and 6.1 ± 2.2 s, 3.4 s, and 10.2 s for the extraverted group. The mean difference of the averaged reaction time between the introversive and extroversive groups is 3.3 s and the statistical testing result (*p* = 0.008) indicates that the difference is significant between the two groups. On the other hand, no statistical significance was observed in the total speech time (*p* = 0.163), averaged pitch frequency (*p* = 0.585), and averaged sound power (*p* = 0.526).

Table 3 shows descriptive statistics for the total number of words, the total number of hedges, total number of mitigators, and total number of high-rising terminals of each participant for the introversive and extroversive groups. The mean ± SD, minimum, and maximum of the total number of words are 35 ± 17 words, 15 words, and 74 words for the introversive group, and 30 ± 15 words, 18 words, and 59 words for the extraverted group. However, no statistical significance was observed in the total number of words (*p* = 0.521), total number of hedges (*p* = 0.828), total number of mitigators (*p* = 0.845), or total number of high-rising terminals (*p* = 0.579).

Binary-logistic regression models have been developed to estimate personality classes (introversive or extroversive) by incorporating each of the significant features (averaged reaction time and total reaction time across all answers). Note that, because other variables including lexical features and some speech features were not significant in the collected datasets, we developed the models using the significant variables (ART and TRT; see Table 2). The Equation (2) shows a binary logistic regression model by using the ART and Equation (3) shows a model by using the TRT. All the *p*-values of the Equations (2) and (3) are less than 0.05 (*p* = 0.029 for the constant term and 0.026 for the averaged reaction time term of the Equation (1), and *p* = 0.027 for the constant term and 0.024 for the total reaction time of the Equation (3)). The classification rule of the developed model depends on the probability estimated by the model, which would be ranged from 0 to 1. If the estimated probability of any given case is greater than 0.5, then the case is classified as an extroversive person. On the other hand, if the estimated probability (P) is less than or equal to 0.5, then the case is classified as an introversive person. The probability of any given case can be calculated by the following Equations (2) and (3):

(2) $$P(\mathrm{averaged}\;\mathrm{reaction}\;\mathrm{time})=\frac{e^{\left(3.645-3.839\;\times \;\mathrm{averaged}\;\mathrm{reaction}\;\mathrm{time}\right)}}{{1+e}^{\left(3.645-3.839\;\times \;\mathrm{averaged}\;\mathrm{reaction}\;\mathrm{time}\right)}}$$

(3) $$P(\mathrm{total}\;\mathrm{reaction}\;\mathrm{time})=\frac{e^{\left(3.677-0.485\;\times \;\mathrm{total}\;\mathrm{reaction}\;\mathrm{time}\right)}}{{1+e}^{\left(3.677-0.485\;\times \;\mathrm{total}\;\mathrm{reaction}\;\mathrm{time}\right)}}$$

Table 4a,b show the confusion matrices of the developed model using averaged reaction time and total reaction time, respectively. The confusion matrix is a useful method for analyzing how well the developed models can recognize the classes. The two models show the same performance and acceptable results, with 70.8% overall classification accuracy. In terms of sensitivity (true positive rate: In this study, it indicates a true extrovert rate predicted by the model) and specificity (true negative rate: In this study, it indicates a true introvert rate predicted by the model), the models show 66.7% sensitivity and 75.0% specificity, which indicates that the developed models perform slightly better in estimating an introvert than an extrovert.

To evaluate the generalizability of the developed classification models, leave-one-out cross-validation (LOOCV) was conducted by using the MATLAB program (R2016b, MathWorks, Inc., Natick, MA, USA). We chose LOOCV method because the size of the datasets (24 participants) is too small to split into training and testing sets; we wanted to use all the data for training, unlike the validation set methods (e.g., 5-fold or 10-fold cross-validation for large sized datasets).

Table 5a,b show the LOOCV results for the averaged and total reaction time models, respectively. The overall classification accuracy of the models was 62.5% with 58.3% sensitivity and 66.7% specificity.

**Figure 2:**
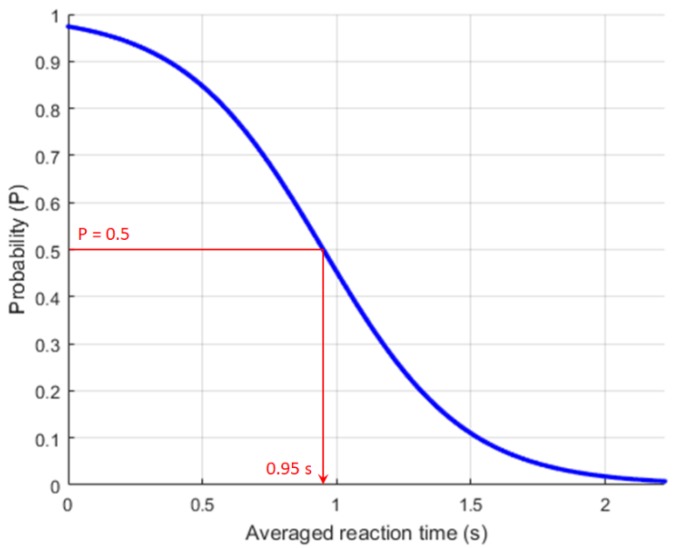
Binary logistic regression model showing the probability of extrovert and introvert as a function of averaged reaction time.

The developed model is particularly useful to quantify a cutoff value to distinguish introvert and extrovert based on an averaged or total reaction time. For example, Figure 2 shows predicted probability (*P*) to classify extrovert (*P* > 0.5) and introvert (*P* ≤ 0.5) by using the developed logistic regression model (Equation 2) as a function of averaged reaction time (unit: second). Based on the coefficients (*β*_0_ = 3.645, *β*_1_ = −3.839) of the classification model for an averaged reaction time, a cutoff is calculated as 0.95 s (= 3.645 divided by 3.839) that indicates 0.5 probability. Therefore, if an averaged reaction time of a human during the human-robot interaction is less than equal to 0.95 s, then the personality of that person would be classified as an extrovert.

## 4. Discussion

The present paper is completely different from the previous proceeding [38] in two major aspects. The first reason is that the present work uses different measures (averaged reaction time, averaged pitch frequency, averaged sound power) to identify two personalities (introversive vs. extroversive), which are never used in the previous study. The second reason is that the present work provides logistical regression models to classify two personalities for the first time. In addition, the present study includes the confusion matrices indicating the model performance in terms of overall and cross-validation accuracy.

Significant speech features between the introversive and extroversive males have been identified. The averaged and total reaction time (all *p*-values are less than 0.05) show significant differences between the introversive males (averaged reaction time = 1.2 s; total reaction time = 9.4 s) and the extroversive males (averaged reaction time = 0.8 s; total reaction time = 6.1 s). The results indicate that introversive males react slowly (0.4 s) compared to extroversive males in responding to a series of questions. This finding corroborates the previous studies which observed that introverts produce longer silence before completing complex verbal tasks ([30,31], among many others). The current study also supports previous assumptions of faster motor processing in extroverts as noted in many brain psychology studies. For example, Stahl and Rammsayer (2008) [39] found that extroverts have faster processing brains, as the pathway of stimuli is much shorter than in introverts’ brains. Brebner and Cooper (1974) [40] asserted that high level excitability of extroverts’ nervous system makes them more susceptible to errors than introverts, but it also accounts for their faster reaction time. Therefore, we can assume that human’s reaction time, rather than total speech time or sound power, is a key attribute that would allow the robot to speculate on the person’s personality (introversive vs. extroversive). However, this assumption needs to be validated by conducting another experiment on human-robot interactions in a future study.

The developed models are particularly useful for a robot to distinguish human personality based on a user’s reaction time during the verbal interaction between a user and a robot. Based on our investigations in this study, we found that the introverts react in average 0.4 s slower than the extroverts, which is usable quantitative information at robots’ disposal in detecting the user personality. However, to accurately recognize human personality during the interaction, the statistical models are vital. The proposed models are easily programmed and embedded in a robot to identify human personality with 70% classification accuracy. Even though the proposed two models have the same classification accuracy, the averaged reaction time model would be more applicable in naturalistic conversation, since the total reaction time model is limited to the sum of reaction times in less than five minutes of speech.

The present study has five limitations. First, in this study, the number of sample size (*n* = 24) is relatively small and the participants are limited to university male students in their 20s. The small number of the male participants creates a weaker data group, which results in a lower statistical power overall. However, we believe that this number of sample size is decent to see the pattern of speech characteristics of different personality groups. We will recruit various participants in terms of gender and age to increase the generalizability of the results for a future report. Second, because we only analyzed male speakers in our datasets, the current result is limited in understanding the gender effects on speech characteristics for personality identification. Although we have some female speech data in our repository, the number of female participants was too small to conduct a statistical testing (*n* = two for the introversive group and one for the extroversive groups). Since the female participants showed relatively high pitch frequency (range = 280–290 Hz) compared to that of the males (range = 130–170 Hz), the participant groups need to be blocked by the gender with appropriate sample sizes. In our future study, we will collect more female speech data to investigate the gender effects. Third, the present study is human-human interaction, not human-robot interaction; although the experimental procedure was carefully designed to remove any unknown factors potentially affecting the data generated from the moderator-participant interaction, it would be imprudent to assert that there are no moderator effects on the answers. In a future study, we aim to use a robot to ask the same questions to participants to identify and mitigate the effects of any confounding factors. Fourth, the recording time in this study is relatively shorter than similar studies conducted in the field of linguistics. Per participant, we collected the verbal answers drawn from an interaction that lasted less than five minutes. Along with the fact that the experiment (involving a preset list of questions) was not designed to elicit naturalistic conversations that would yield a full spectrum of linguistic features, we are aware that the dataset we examined is insufficient in identifying significant lexical and dialogic patterns in this such a short time (5 min). For a future experiment, we are currently planning on increasing the data recording time, as well as modifying the question prompts to see more interactional patterns between robots and humans with the human personality as a variable. Lastly, the responses could be affected by the other three letters (sensing/intuition, thinking/feeling, and judging/perception) in MBTI personality classes. In this study, we focused only on two classes (introversive vs. extroversive) while blocking the effects of other personality dimensions. It is possible that the other traits, as well as the degree of each trait, might affect the output during an interaction. In a future study, we may need to select participants more carefully by considering all four letters.

Moving forward, more studies that investigate the human-robot interaction are called for in order to validate certain associations between personality traits and speech characteristics (e.g., extroverted-energetic voice, introverted-subdued voice). Considering other behavioral characteristics such as facial recognition or gaze/gesture behavior would also benefit the effort to more accurately capture human personality.

## 5. Conclusions

The present study identified significant speech characteristics between 12 introvert and 12 extrovert males. The averaged reaction time shows significant difference (*p* = 0.010) between introverts (Mean = 1.2 s, SD = 0.4) and extroverts (Mean = 0.8 s, SD = 0.3). The total reaction time shows significant difference (*p* = 0.008) between introverts (Mean = 9.4 s, SD = 3.1) and extroverts (Mean = 6.1 s, SD = 2.2). Upon these findings, the present study proposes binary logistic regression models to seamlessly identify human personality based on the averaged and total reaction time. The two proposed models show an overall 70% classification accuracy (66.7% sensitivity and 75.0% specificity). These results would be particularly useful for a robot to estimate a user’s personality, which would then allow a robot to react accordingly and agreeably to the estimated personality. Since the sample size is too small to have strong statistical power, however, the greater number of observations and longer conversational datasets should be obtained in the future work, especially for identifying a wider range of linguistic features. Nevertheless, we believe that the results in this study warrant several larger studies in the future.

## Figures and Tables

**Table 1:** The MBTI personality classes of the participants in this study.

No.	Introversive Group (*n* = 12)	Extroversive Group (*n* = 12)
1	ISTJ	ESTJ
2	ISTJ	ESTJ
3	ISTP	ESTP
4	ISFJ	ESTP
5	INTJ	ESFJ
6	INTJ	ESFP
7	INTP	ENTJ
8	INFJ	ENTP
9	INFP	ENTP
10	INFP	ENFJ
11	INFP	ENFJ
12	INFP	ENFJ

Note: The four letters represent the traits of personality, which describe Mind (Introversive or Extroversive), nature (iNtuition or Sensing), energy (Thinking or Feeling), and tactics (Perception or Judging).

**Table 2:** Descriptive statistics of the speech and sound features of the introversive and extroversive groups.

Speech Features	Introversive Group (*n* = 12)				Extroversive Group (*n* = 12)				Statistics	
	Mean	SD	Min	Max	Mean	SD	Min	Max	***t***	***p***
ART (s)	1.2	0.4	0.6	2.2	0.8	0.3	0.4	1.3	−2.87	0.010
TRT (s)	9.4	3.1	4.5	15.6	6.1	2.2	3.4	10.2	−2.95	0.008
TST (s)	10.7	4.5	4.9	20.8	8.2	3.8	3.5	16.6	−1.45	0.163
APF (Hz)	118	15	102	152	114	16	94	138	−0.55	0.585
ASP (W)	0.001	0.001	0.000	0.003	0.001	0.001	0.000	0.005	0.65	0.526

Note: ART = averaged reaction time (unit: second), TRT = total reaction time (unit: second), TST = total speech time (unit: second), APF = averaged pitch frequency (unit: Hz), ASP = averaged sound power (unit: Watt), SD = standard deviation.

**Table 3:** Descriptive statistics of the lexical features of the introversive and extroversive groups.

Lexical Features	Introversive Group (*n* = 12)				Extroversive Group (*n* = 11)				Statistics	
	Mean	SD	Min	Max	Mean	SD	Min	Max	***t***	***p***
TNW	35	17	15	74	30	15	18	59	0.65	0.521
TNH	2	2	0	5	2	2	0	5	−0.22	0.828
TNM	1	2	0	6	1	2	0	6	0.20	0.845
TNHRT	1	1	0	2	1	1	0	3	−0.56	0.579

Note: TNW = total number of words, TNH = total number of hedges, TNM = total number of mitigators, TNHRT = total number of high-rising terminals, SD = standard deviation.

**Table 4:** Classification results of the developed binary logistic models.

(**a**) Confusion matrix with the averaged reaction time model.				
		Actual		
		Extrovert	Introvert	Total
Predicted	Extrovert	8	3	11
	Introvert	4	9	13
	Total	12	12	24
(**b**) Confusion matrix with the total reaction time model.				
		Actual		
		Extrovert	Introvert	Total
Predicted	Extrovert	8	3	11
	Introvert	4	9	13
	Total	12	12	24

**Table 5:** Validation results of the developed binary logistic models.

(**a**) Confusion matrix with the averaged reaction time model.				
		Actual		
		Extrovert	Introvert	Total
Predicted	Extrovert	7	4	11
	Introvert	5	8	13
	Total	12	12	24
(**b**) Confusion matrix with the total reaction time model.				
		Actual		
		Extrovert	Introvert	Total
Predicted	Extrovert	7	4	11
	Introvert	5	8	13
	Total	12	12	24
